# Supplementary material for: Rapid Increase in the IS26-Mediated cfr Gene in E. coli Isolates with IncP and IncX4 Plasmids and Co-Existing cfr and mcr-1 Genes in a Swine Farm
Source: Pathogens. 2021 Jan 3;10(1):33. doi: 10.3390/pathogens10010033 (PMC7823714; doi:10.3390/pathogens10010033)
Supplement: Supplementary file 1 [file pathogens-10-00033-s001.pdf]

SUPPLEMETAL MATERIALS

Table 1. Characterization of *cfr*-carrying transformants.

Isolates	MIC values [a]				Replicon type	Resistance genes
	AMP	FLR	CL	APR		
GDE5P101T	1	4	4	4	IncI2	*cfr mcr-1*
GDE5P132T	2	2	4	4	IncI2	*cfr mcr-1*
GDE5P137T	128	2	4	128	IncX4	*cfr mcr-1*
GDE6P124T	128	4	4	4	IncP	*cfr mcr-1*
GDE6P128T	128	4	4	4	IncP	*cfr mcr-1*
GDE6P129T	2	4	4	4	IncX4	*cfr mcr-1*
GDE6P133T	2	2	4	4	IncX4	*cfr mcr-1*
GDE6P143T	2	2	4	4	IncX4	*cfr mcr-1*
GDE6P164T	4	2	2	4	IncX4	*cfr mcr-1*
GDE6P165T	4	2	2	4	IncX4	*cfr mcr-1*
GDE6P168T	128	4	4	4	IncX4	*cfr mcr-1*
GDE6P169T	128	4	4	4	-	*cfr mcr-1*
DH5α	2	2	0.125	4	-	-
ATCC 25922	2	2	0.5	2	-	-

Note: a. AMP: ampicillin; APR: apramycin; FLR: florfenicol; CL: colistin.

Table S2. information of sample collection in the swine farm during the period of 2014-2017.

Collected date	Suckling piglet (N)	Nursery pig (N)	Fattening pig (N)	Sow (N)	Boar (N)	Environment (N)	Total
Jun-2014	50	40	72	57	0	0	220
Jul-2015	43	80	67	51	0	0	241
Jul-2016	15	55	56	60	10	4	200
Apr-2017	15	50	60	55	10	10	200
Total	123	226	255	223	20	14	861

Table 3. Primers used for PCR and DNA sequencing in this study.

Genes	Prime Sequence (5′ to 3′)	Size	References
cfr	F:TGAAGTATAAAGCAGGTTGGGAGTCA<br>R:ACCATATAATTGACCACAAGCAGC	746	[1]
floR	F:CTGAGGGTGTCGTCATCTAC<br>R:GCTCCGACAATGCTGACTAT	673	[2]
fexA	F:GTACTTGTAGGTGCAATTACGGCTGA<br>R:CGCATCTGAGTAGGACATAGCGTC	977	[1]
fexB	F:TTCCCACTATTGGTGAAAGGAT<br>R:GCAATTCCCTTTTATGGACGTT	750	[3]
optrA	F:AGGTGGTCAGCGAACTAA<br>R:ATCAACTGTTCCCATTCA	1395	[4]
mcr-1	F:TCGCGGCATTCGTTATA<br>R:GGTGGCGTTCAGCAGTC	582	[5]
parA of IncP	F:AGTGTATTTGCGAGTTAG<br>R:GATGCGTTTATGAAGC	496	In this study
IS*26*-*cfr*	F:CGTTCAGCCAGCATCT<br>R:TCGCCTGTAGCACAAA	663	In this study
cfr-IS*26*	F:AAAGCAGGTTGGGAGTC<br>R:GTGAAGAAGTGGCAGA	1207	In this study

Note: F: forward primer; R: reverse primer.

References:

[1] Kehrenberg, C, Schwarz, S. Distribution of florfenicol resistance genes fexA and cfr among chloramphenicol-resistant Staphylococcus isolates. *Antimicrob Agents Chemother* 2006; 50: 1156-63.

[2] Chen S, Zhao S, White DG *et al*. Characterization of multiple-antimicrobial-resistant *salmonella* serovars isolated from retail meats. *Appl Environ Microbiol* 2004; 70:1-7.

[3] Liu HB, Wang Y, Wu CM et al. novel phenicol exporter gene, fexB, found in enterococci of animal origin. J Antimicrob Chemother 2012; 67: 322-5.

[4] Wang Y, Lv Y, Cai J *et al.* A novel gene, *optrA*, that confers transferable resistance to oxazolidinones and phenicols and its presence in *Enterococcus faecalis* and *Enterococcus faecium* of human and animal origin. *J Antimicrob Chemother* 2015; 70:2182-90.

[5] Wang J, Huang XY, Xia YB *et al*. Clonal Spread of Escherichia coli ST93 Carrying *mcr-1*-Harboring IncN1-IncHI2/ST3 Plasmid Among Companion Animals, China. *Front Microbiol* 2018; 9:2989.
